# Factor H-related protein 1 promotes complement-mediated opsonization of *Pseudomonas aeruginosa*

**DOI:** 10.3389/fcimb.2024.1328185

**Published:** 2024-03-06

**Authors:** Alex González-Alsina, Héctor Martín-Merinero, Margalida Mateu-Borrás, María Verd, Antonio Doménech-Sánchez, Joanna B. Goldberg, Santiago Rodríguez de Córdoba, Sebastián Albertí

**Affiliations:** ^1^ Instituto Universitario de Investigación en Ciencias de la Salud (IUNICS), Universidad de las Islas Baleares and Instituto de Investigación Sanitaria de les Illes Balears (IDISBA), Palma de Mallorca, Spain; ^2^ Center for Biological Research-Margarita Salas and Centro Investigación Biomédica En Red (CIBER) de Enfermedades Raras, Madrid, Spain; ^3^ Department of Pediatrics, Emory University School of Medicine, Atlanta, GA, United States

**Keywords:** FHR-1, factor H, complement system, *P. aeruginosa*, OprG

## Abstract

*Pseudomonas aeruginosa* is an important human opportunistic pathogen responsible for a wide range of infections. The complement system is the main early host defense mechanism to control these infections. *P. aeruginosa* counteracts complement attack by binding Factor H (FH), a complement regulator that inactivates C3b, preventing the formation of the C3-convertase and complement amplification on the bacterial surface. Factor H-related proteins (FHRs) are a group of plasma proteins evolutionarily related to FH that have been postulated to interfere in this bacterial mechanism of resisting complement. Here, we show that FHR-1 binds to *P. aeruginosa* via the outer membrane protein OprG in a lipopolysaccharide (LPS) O antigen-dependent manner. Binding assays with purified components or with FHR-1-deficient serum supplemented with FHR-1 show that FHR-1 competes with FH for binding to *P. aeruginosa.* Blockage of FH binding to C3b deposited on the bacteria reduces FH-mediated cofactor activity of C3b degradation, increasing the opsonization of the bacteria and the formation of the potent chemoattractant C5a. Overall, our findings indicate that FHR-1 is a host factor that promotes complement activation, facilitating clearance of *P. aeruginosa* by opsonophagocytosis.

## Introduction

*Pseudomonas aeruginosa* is an important human opportunistic pathogen responsible for a wide variety of infections including keratitis, acute and chronic respiratory infections as well as bloodstream infections. Upon infection of the host, *P. aeruginosa* is rapidly attacked by early innate immune effectors such as the complement system. Much experimental evidence supports the essential role of complement to control *P. aeruginosa* infections. Early studies demonstrated that complement-deficient mice were impaired in their capacity to clear *P. aeruginosa* from their corneas or lungs (Cleveland et al., 1983; Mueller-Ortiz et al., 2004). More recently, Pont et al. demonstrated that, in human blood, complement is the main defensive mechanism against *P. aeruginosa* (Pont et al., 2020).

The complement system is activated by three pathways; the classical, the lectin, and the alternative pathway. Activation by any of these pathways results in the formation of the enzyme C3 convertase, which cleaves the central component of the complement system, C3, into surface-opsonizing C3b and into the anaphylatoxin and chemotactic molecule C3a. If activation continues, C3b can bind to C3 convertases to generate the C5 convertases. These new enzyme complexes hydrolyze C5 molecules, producing the potent chemoattractant peptide C5a and C5b, which will proceed to form a membrane attack complex on the bacterial surface. Thus, complement system may lyse directly the bacterial cells, opsonize them to facilitate the recognition by the phagocyte cells, and release anaphylatoxins that promote phagocyte recruitment and pro-inflammatory state at the site of infection.

*P. aeruginosa*, as many other microbial pathogens, has evolved different strategies to counteract the effects of the complement system (González-Alsina et al., 2023). One of these strategies relies on the binding of host factors that inhibit complement activation, such as Factor H (FH) and the Factor H-like protein 1 (FHL-1) (Józsi, 2017; Kunert et al., 2007; Hallström et al., 2012). These regulatory proteins compete with Factor B for the binding to C3b and inhibit the formation of the alternative pathway C3 convertase by acting as a cofactor for Factor I degrading C3b into inactive C3b (iC3b) or accelerating the decay of the convertase (Józsi, 2017).

FH is a 155-kDa circulating plasma glycoprotein that consists of 20 short consensus repeats (SCR) domains, while FHL-1 is derived from an alternative splicing of the *CFH* gene, giving rise to a protein of 42 kDa containing SCR 1-7 (**Supplementary Figure 1**). The complement regulatory region is located in the N-terminal region (SCR 1-4) of both proteins (Zipfel et al., 1999). In humans, the cluster that encodes for the *CFH* gene is located in chromosome 1 and includes five additional genes which encode complement FH-related proteins 1-5 (FHR-1 to FHR-5). FHR-1 is the most abundant protein in plasma among FHR proteins. It is composed of only five SCRs that are homologous to SCRs 6 -7 and 18-20 of FH (Zipfel et al., 1999; Józsi et al., 2015) (**Supplementary Figure 1**). Two FHR-1 glycoforms, FHR-1α (37 kDa) and FHR-1β (43 kDa) are detected in plasma, representing differentially glycosylated proteins. FHR-1 cannot regulate the complement system but act as deregulator of the complement since they compete with FH for ligand binding due to their similar structure (Józsi et al., 2015).

*P. aeruginosa* binds FHR-1 through the Elongation Factor Tu (EF-Tu) and the dihydrolipoamide dehydrogenase (LPD) (Kunert et al., 2007; Hallström et al., 2012). However, the biological consequences of these interactions remain poorly investigated. In this work, we have investigated the interaction of FHR-1 with this microorganism and the biological consequences in the pathogenesis of *P. aeruginosa* infections.

## Materials and methods

### Bacterial strains

*P. aeruginosa* reference strain PAO1 and its isogenic *wzz1*-deficient mutant (PAO1Δ*wzz1*), *wzz2*-deficient mutant (PAO1Δ*wzz2*), and *galU*-deficient mutant (PAO1Δ*galU*), were previously described (Held et al., 2012; Dean and Goldberg, 2002). *wzz1* and *wzz2* encode LPS O antigen chain length regulators. These genes control the expression of long and very long chain lengths, respectively. Interruption of *galU* gene results in production of LPS devoid of O antigen (rough LPS) and truncated LPS core. *P. aeruginosa* strain H899 (wild-type PAO1) and the isogenic *oprG*-deficient mutant (H900) were previously described (McPhee et al., 2009). *Escherichia coli* strain S17-λpir was used in the cloning experiments.

Bacterial cells were grown in Luria Bertani (LB) broth at 37°C with shaking or in LB solidified with 1.5% agar. In the cloning experiments, LB was supplemented with ampicillin (50 µg/ml) or carbenicillin (250 µg/ml).

### Human serum and proteins

Blood samples were collected from five healthy individuals or from two donors with FHR-1/FHR-3 deficiency. Blood was coagulated at 37°C for 30 min and then centrifuged at 4,500 x g for 20 min at 4°C to separate serum. Equal volumes of serum from each healthy individual or from the two donors with FHR-1/FHR-3 deficiency were mixed to get a pool of normal human serum (NHS) or a pool of FHR-1-deficient human sera (ΔFHR-1) that was aliquoted and stored at −80°C until its use. In some experiments, sera were heat inactivated at 56°C for 30 min or with EDTA (5 mM) prior to use.

Recombinant human FHR-1 was produced, purified, and stored as described previously (Martin Merinero et al., 2021). Purified human FH was obtained from HycultBiotech.

### FH and FHR-1 binding assays

The binding of FH or FHR-1 to bacterial cells was determined by ELISA. Briefly, 96-well round-bottom polystyrene microtiter plates wells were coated overnight at 37°C with 10^8^ bacterial cells resuspended in phosphate-buffered saline (PBS). Next, wells were blocked with PBS containing 1% bovine serum albumin (PBS-BSA) and incubated with PBS as control, FH (20 µg/ml), FHR-1 protein (at different concentrations depending on the experiment) or human sera (10%) diluted in PBS. FH and FHR-1 were detected with the specific mouse monoclonal antibodies OX-24 (Abcam) and MBC125 (Tortajada et al., 2017), respectively. Finally, wells were incubated with an alkaline phosphatase-conjugated goat anti-mouse immunoglobulin G (Sigma), and developed with p-nitrophenyl phosphate (Sigma) in 50 mM carbonate-bicarbonate buffer, pH 9.6, 5mM MgCl_2_. Absorbance was measured at 415 nm. Controls values were < 0.1 optical density units and were subtracted from the experimental values. Washing steps with PBS were included between incubations that were performed for 1 h at 37°C.

### Isolation, analysis, and identification of outer membrane proteins

Outer membrane proteins were isolated as previously described with some modifications (Qadi et al., 2017). Briefly, bacterial cells (1 x 10^10^) were lysed by sonication and cell envelopes were isolated by centrifugation at 14,000 x *g* for 30 min at 4°C. Outer membrane proteins were isolated from cell envelopes as sodium lauryl sarcosinate–insoluble material by centrifugation at 14,000 x *g* for 30 min at 22°C. For the analysis, the outer membrane preparations were resuspended in sample buffer (50 mM Tris-HCl, pH 6.8, 2% Sodium Dodecyl Sulphate (SDS), 10% glycerol, 0.01% bromophenol blue), boiled for 5 minutes, resolved by Sodium Dodecyl Sulphate-Polyacrylamide gel electrophoresis (SDS-PAGE), and visualized by Coomassie blue staining. Selected protein spots were excised from the gels, trypsin digested, and identified by tandem mass spectrometry as described elsewhere (Barbier et al., 2013).

### Western blot analysis

Binding of FHR-1 to *P. aeruginosa* was analyzed by Western blot. For the binding of FHR-1, 1x10^10^ bacterial cells were incubated in normal human serum (NHS) at 50% in PBS with EDTA (5 mM) for 1 h at 37°C. After incubation, bacteria were exhaustively washed with PBS. Bound FHR-1 was eluted with 0.1 M glycine-HCL (pH 2.0), mixed with sample buffer (see above) and subjected to Western blot analysis using the monoclonal antibody MBC125 to identify FHR-1. Membranes were scanned with a Bio-Image densitometer (Millipore).

### C3 binding assays

Binding of C3 to bacterial cells was determined by ELISA as previously described (Qadi et al., 2017). Bacterial cells (1x10^9^ CFU) were washed with PBS and incubated for 15 min at 37°C with human serum (20%) supplemented or not with FHR-1 (20 µg/ml). HI-serum was used as control. Next, bacteria were washed and incubated for 2 h at 37°C in 50 mM carbonate-bicarbonate buffer (pH 9.0) containing 1 M NH_4_OH to disrupt ester bonds between C3 fragments and the bacterial surface. Serial dilutions of the eluted cell-bound C3 were used to coat microtiter plate wells at 4°C for 18 h. Wells were blocked with PBS-BSA, incubated sequentially with a rabbit polyclonal anti-human C3 (Abcam, ab117244) which detects C3, C3b and iC3b, and alkaline phosphatase-labeled goat anti-rabbit immunoglobulin G (Sigma), and developed with p-nitrophenyl phosphate (Sigma) in 50 mM carbonate-bicarbonate buffer (pH 9.6) plus 5 mM MgCl_2_. Controls values were < 0.1 optical density units and were subtracted from the experimental values.

### DNA procedures

For the cloning of *oprG*, the gene was amplified by PCR from the genome of PAO1 using primers OPRGF1 (5′-CCCCCCGAATTCAGCTCATCATGCGT-3′) and OPRGR2 (5′-GGGGGGAAGCTTTACGACTCAGAATTGTAGGCC-3′), containing EcoRI and HindIII sites, respectively. The resulting PCR product was digested with both enzymes and cloned into pJPO4 (Owings et al., 2016) to give plasmid pMMB2. Plasmid pMMB2 was transformed into *P. aeruginosa* or *E. coli* S17-λpir by electroporation. All molecular biology techniques were performed according to standard protocols as described previously (Sambrook et al., 1989).

### C5a quantification

Bacterial cells (1x10^9^) of *P. aeruginosa* were incubated for 15 min at 37°C in FHR-1-deficient human sera (ΔFHR-1) (25%) diluted in PBS supplemented with FHR-1 (25 µg/ml) or not. Human serum incubated with FHR-1 without bacteria was used as negative control. After incubation, bacterial suspension was centrifuged and the amount of C5a present in the supernatant was quantified using the Human C5a/Complement C5a ELISA Kit (Sigma-Aldrich) following the manufacturer’s instructions.

### Ethics statement

All human samples were taken after obtaining the “Informed consent” of the participants. They had been informed of the purposes of the study. The study was reviewed and approved by the Ethics Review Committee of Human Experimentation of Islas Baleares [Comité de ética de la Investigación de las Islas Baleares (CEI-IB)].

All study procedures were performed in accordance with relevant standard international guidelines/regulations.

### Statistical analysis

The statistical significance of the data obtained in at least three independent experiments was determined by ANOVA with *post hoc* Tukey. In all cases, a p value of <0.05 was considered statistically significant. Statistical analyses were performed using IBM SPSS Statistics v22 software.

## Results

### LPS O antigen modulates FHR-1 binding to *P. aeruginosa*

It has been reported that *P. aeruginosa* clinical isolates from different origins bind FHR-1 (Kunert et al., 2007). However, the bacterial factors that modulate this binding are unknown. LPS is a major complement resistance factor that blocks the binding of complement components to *P. aeruginosa.* We used a set of isogenic LPS mutants deficient in the long LPS O antigen side chain (PAO1Δ*wzz1*), the very long LPS O antigen side chain (PAO1Δ*wzz2*) and LPS devoid of O antigen (PAO1Δ*galU*) to investigate the role of this critical virulence factor on the binding of FHR-1 to *P. aeruginosa*. PAO1Δ*wzz1* lacks the long O side chain of the LPS but possesses a very long LPS O antigen side chain. On the other hand, PAO1Δ*wzz2* lacks the very long O antigen side chains but has long LPS O chains. Consequently, PAO1Δ*wzz2* exhibits shorter O antigen side chains compared to PAO1Δ*wzz1*.

Microtiter ELISA plate wells coated with bacterial cells were incubated with purified human FHR-1 or normal human serum (NHS); the amount of FHR-1 bound to the bacterial cell was detected with a specific monoclonal antibody that recognizes FHR-1.

**Figure 1:**
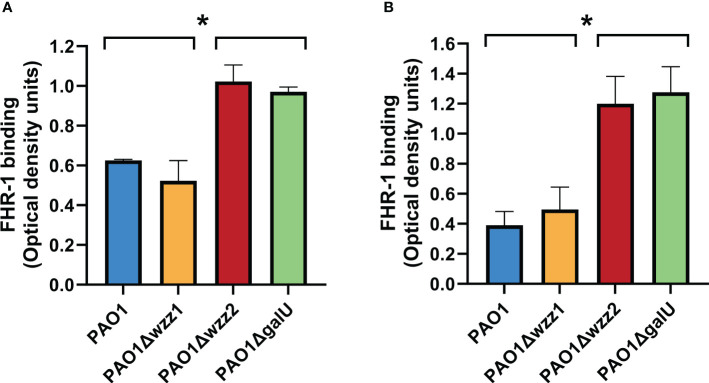
LPS O antigen length modulates FHR-1 binding to *P. aeruginosa.* The binding of FHR-1 to a set of isogenic LPS-deficient strains incubated with purified FHR-1 **(A)** or in NHS **(B)** was analyzed by whole-cell ELISA. Bound FHR-1 was detected with a specific monoclonal antibody. Bars represents the mean of at least three independent experiments done in duplicate, and SD is indicated by error bars. Statistical analyses were performed using ANOVA with *post hoc* Tukey; *P < 0.05.

We observed that the serum-sensitive strains PAO1Δ*wzz2* and PAO1Δ*galU* acquired more FHR-1 than the serum-resistant strains PAO1 and PAO1Δ*wzz1*, either incubated with purified FHR-1 or in NHS (**Figure 1**). This result suggests that LPS O side antigen blocks the interaction of FHR-1 with its target/s on the bacterial surface.

### OprG is a FHR-1-binding molecule of *P. aeruginosa*

**Figure 2:**
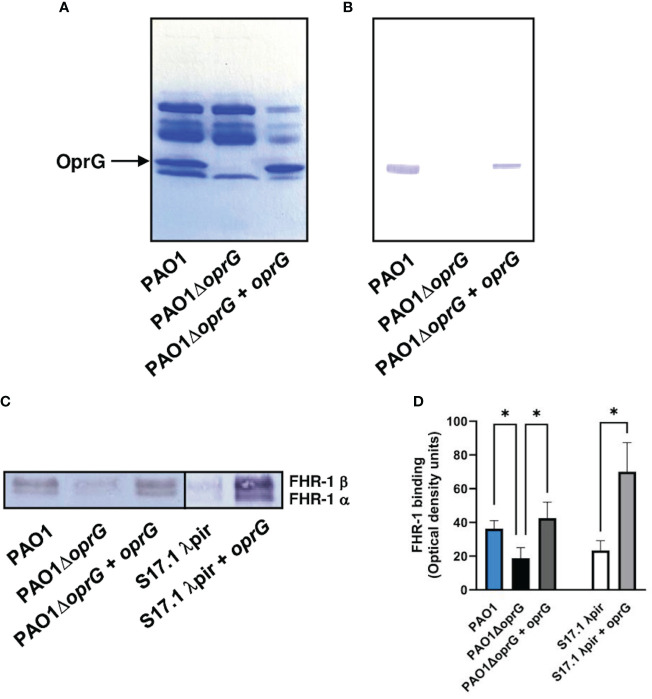
P*. aeruginosa* binds FHR-1 by interacting with the outer membrane protein OprG. Outer membrane proteins from PAO1, its derived OprG-deficient mutant, and the mutant complemented with *oprG* were isolated, resolved and stained with Coomassie blue **(A)** or transferred to a membrane and incubated with FHR-1 **(B)**. Bound FHR-1 was detected with a specific monoclonal antibody. A band of approximately 25 kDa, identified by mass spectrometry as OprG, reacted with FHR-1. Bacterial cells of the *P. aeruginosa* strain PAO1, its derived OprG-deficient mutant (PAO1Δ*oprG*), the mutant complemented with *oprG* (PAO1Δ*oprG* + *oprG), E. coli* strain S17.1 λpir, and *E. coli* complemented with *oprG (*S17.1 λpir + *oprG)* were incubated with NHS- EDTA. The proteins bound to the bacteria were eluted, separated by SDS-PAGE and subjected to a Western blot using the mouse monoclonal antibody that recognizes FHR-1, MBC125 **(C)**. Densitometric analysis of FHR-1 bands **(D)**. Bars represents the mean of at least three independent experiments done in duplicate, and SD is indicated by error bars. Statistical analyses were performed using ANOVA with *post hoc* Tukey; *P < 0.05.

To identify the surface components involved in the binding of FHR-1 to *P. aeruginosa*, we performed a Western blot analysis of the outer membrane proteins isolated from the strain PAO1 using FHR-1 as probe. One protein of approximately 25-kDa reacted with FHR-1 (**Figures 2A, B**). The corresponding band was excised from the gel and the protein was subjected to mass spectrometry analysis. The band was found to correspond to the outer membrane protein OprG. This result was further confirmed by Western blot analysis of the outer membrane proteins of the isogenic OprG-deficient mutant PAO1Δ*oprG* and the complemented mutant (PAO1Δ*oprG*+*oprG*) (**Figures 2A, B**). FHR-1 did not react with any of the outer membrane proteins isolated from the OprG-deficient mutant, but it reacted with OprG from the complemented mutant.

In order to evaluate the contribution of OprG to the binding of FHR-1 to *P. aeruginosa*, we determined the acquisition of serum FHR-1 by *P. aeruginosa* intact cells. Bacteria were incubated with NHS-EDTA and after exhaustive washing, bound FHR-1 was eluted and analyzed by Western blot with the FHR-1 specific monoclonal antibody (**Figure 2C**). Densitometric analysis of the FHR-1 bands demonstrated that the *oprG* deletion mutant showed significantly decreased FHR-1 binding compared to the wild-type strain and the complemented mutant (**Figure 2D**). To further confirm that OprG is involved in the acquisition of FHR-1 by the bacterial cell, we cloned the *P. aeruginosa oprG* gene in *E. coli.* The presence of OprG in the outer membrane of *E. coli* was confirmed by SDS-PAGE analysis of the outer membrane proteins (**Supplementary Figure 2**). As it is shown in **Figures 2C, D**, the OprG-expressing *E. coli* strain bound higher amount of FHR-1 than the parental strain.

### FHR-1 competes with FH for the binding to *P. aeruginosa*

**Figure 3:**
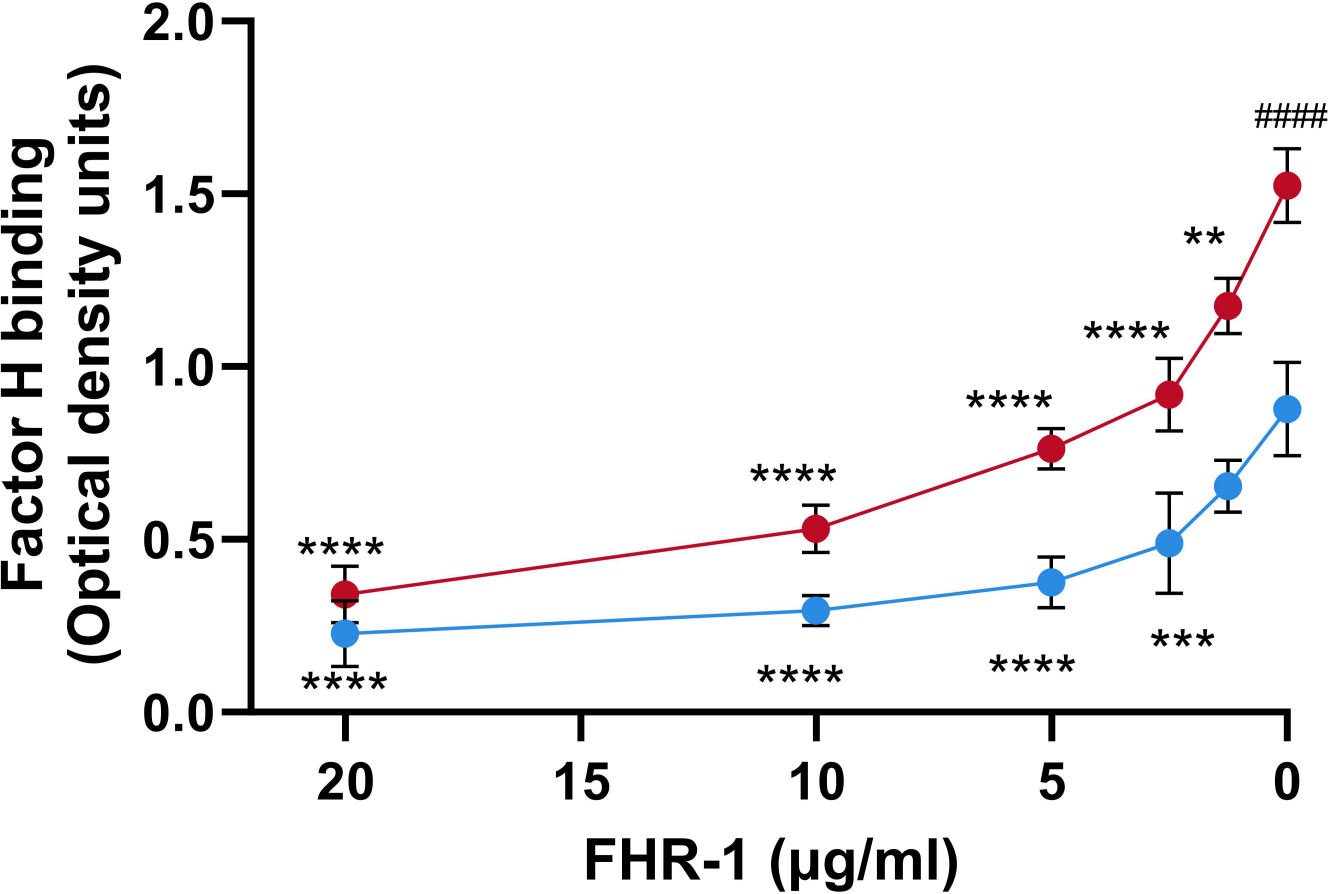
FHR-1 competes with FH for the binding to *P. aeruginosa.* Whole cell-ELISA binding assays of purified FH (20 µg/ml) to the serum-resistant strain PAO1 (blue circles) and the isogenic serum-sensitive LPS-deficient strain PAO1Δ*wzz2* (red circles) in the presence of decreasing concentrations of FHR-1. Bound FH was detected with the FH-specific monoclonal antibody OX-24. Data represent at least three experiments done in duplicate, and SD is indicated by error bars. Asterisks indicate significant differences in FH binding between the absence of FHR-1 and the corresponding FHR-1 concentration. Hashtag indicates significant differences in FH binding between the both strains in the absence of FHR-1. Statistical analyses were performed using ANOVA with *post hoc* Tukey; **P<0.01, ***P<0.001, ****P<0.0001, ^####^ P<0.0001.

It has been suggested that FHRs proteins act as decoys that displace FH from the surface of the pathogen due to their overlapping ligand spectrum with this complement inhibitor (Skerka et al., 2013). To investigate whether FHR-1 is an antagonist of FH for the binding to *P. aeruginosa*, we conducted competition experiments with both purified complement components using the strain PAO1, as a model of serum-resistant strain and the isogenic strain PAO1Δ*wzz2*, as a model of serum-sensitive. Bacterial cells were incubated with purified FH and decreasing amounts of recombinant FHR-1, and the binding of FH to the bacterial surface was determined using the FH-specific monoclonal antibody, OX-24. FHR-1 reduced the amount of FH bound to *P. aeruginosa* PAO1 and PAO1Δ*wzz2* in a dose-dependent manner (**Figure 3**). When the amount of recombinant FHR-1 and FH were at approximately equimolar ratios to those in normal human serum (FH: FHR-1 of 1:0.27) (Skerka et al., 2013), FH binding was approximately 40% and 50% of the level observed in the absence of FHR-1 for PAO1 and PAO1Δwzz2, respectively.

### Biological significance of FHR-1 binding to *P. aeruginosa*

**Figure 4:**
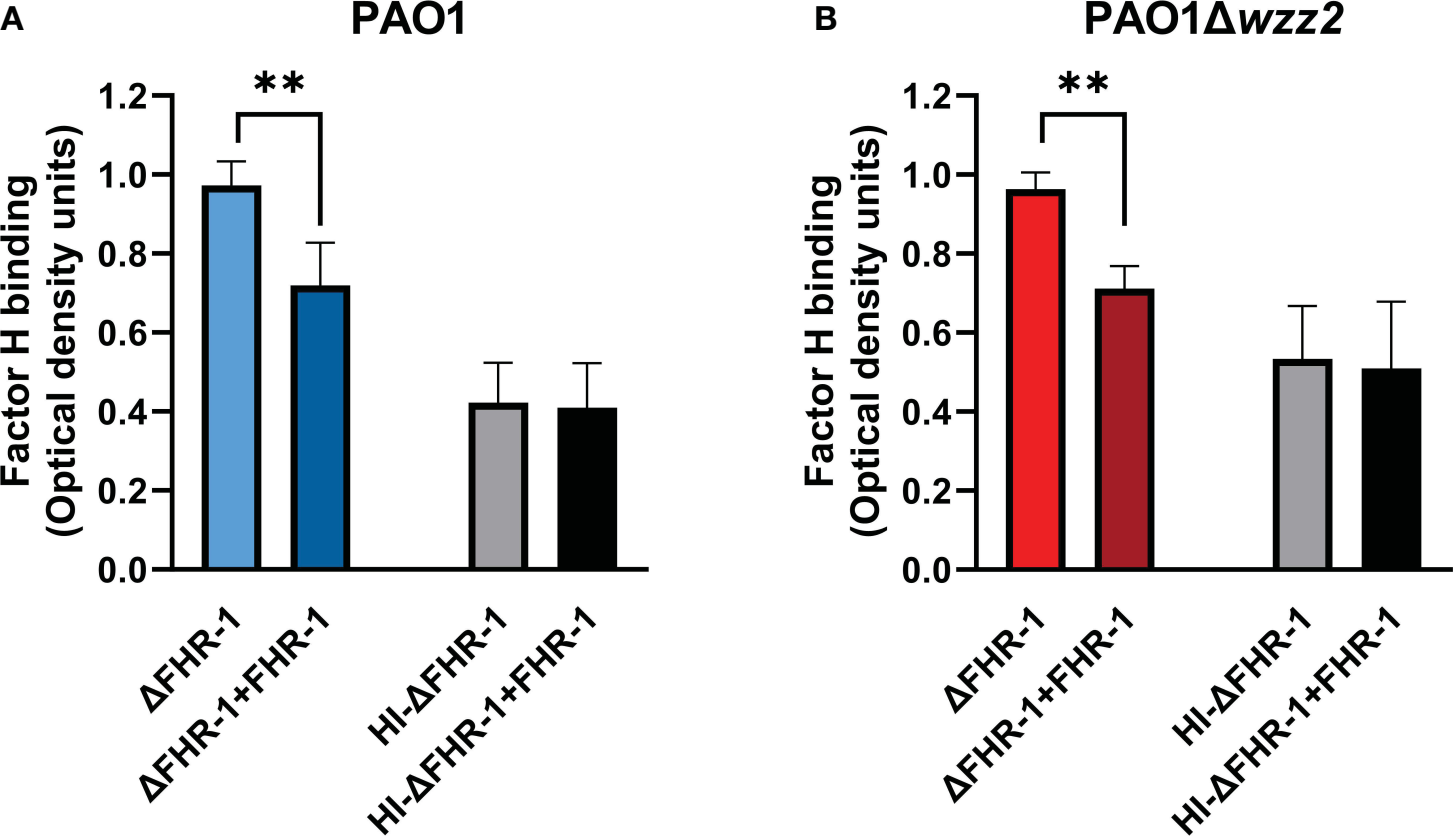
FHR-1 reduces the binding of FH to *P. aeruginosa* in human serum. ELISA binding assays of FH to the serum-resistant strain PAO1 **(A)** or the isogenic LPS-deficient serum-sensitive strain PAO1Δ*wzz2* **(B)** incubated in FHR-1-deficient serum (ΔFHR-1) (10%) supplemented or not with FHR-1 (10 µg/ml). FH was detected with the specific monoclonal antibody OX-24. Bars represents the mean of at least three independent experiments done in duplicate, and SD is indicated by error bars. Statistical analyses were performed using ANOVA with *post hoc* Tukey; **P<0.01.

The effect of FHR-1 on the binding of FH to *P. aeruginosa* was also evaluated using FHR-1-deficient human sera (ΔFHR-1) supplemented or not with equimolar concentrations of FHR-1. Addition of FHR-1 reduced the acquisition of FH around 20-30% by *P. aeruginosa* independently of the strain tested (**Figure 4**). It is remarkable that FHR-1 did not affect the binding of FH to *P. aeruginosa* when human serum was heat-inactivated (HI- ΔFHR-1), suggesting that the inhibition of the binding of FH to the bacteria by FHR-1 may be predominately attributed to the competition of both, FH and FHR-1 for the binding to C3b, the natural ligand of both proteins, rather than to the competition for to the binding to their bacterial targets.

Next, we wanted to evaluate whether the FHR-1-mediated reduction of FH binding to the bacteria had any impact on the deposition of C3 on the bacterial surface. Thus, we incubated the bacteria in ΔFHR-1 serum supplemented or not with FHR-1 and the deposition of C3 on the bacteria was analyzed by ELISA using a polyclonal anti-C3 antibody.

**Figure 5:**
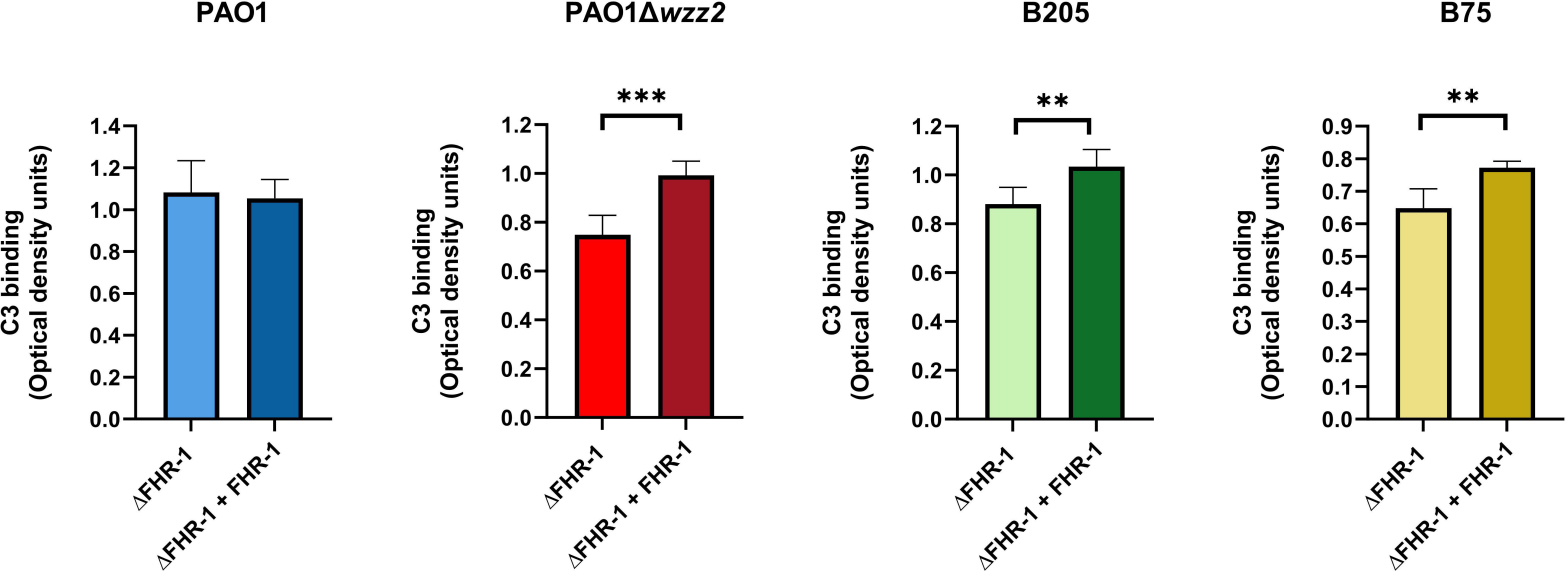
FHR-1 promotes *P. aeruginosa* opsonization. Bacterial cells of the *P. aeruginosa* serum-resistant strain PAO1, the isogenic serum-sensitive mutant PAO1Δ*wzz2* and the clinical isolates B205 and B75 were incubated for 15 min at 37°C in FHR-1-deficient human sera (ΔFHR-1) (20%) supplemented or not with FHR-1 (20 µg/ml). Deposition of C3 on the bacterial surface was determined by ELISA. Bars represents the mean of at least three independent experiments done in duplicate, and SD is indicated by error bars. Statistical analyses were performed using two-tailed t test; *P* values are indicated on the bars; *P < 0.05, **P <0.01, ***P <0.001.

In experiments performed side-by side, the serum susceptible strain PAO1Δ*wzz2*, when incubated in ΔFHR-1 serum, bound more C3 than the parental serum-resistant strain PAO1, which is consistent with their respective resistance phenotype (data not shown). The serum susceptible strain PAO1Δ*wzz2* incubated in ΔFHR-1 serum supplemented with FHR-1 bound more C3 than in its absence (**Figure 5**). By contrast, FHR-1 had no effect on the opsonization of the serum resistant strain PAO1. To emphasize the role of FHR-1 in enhancing C3 deposition on *P. aeruginosa*, we investigated its impact on C3 binding for two clinical strains of *P. aeruginosa*: the serum-sensitive isolate B75 and the serum-resistant isolate B205. These strains were obtained from different patients with bloodstream infections. Consistent with our observations with PAO1 and PAO1Δwzz2, the quantification of C3 fragments (C3b and iC3b) on the bacterial surface revealed elevated levels of C3 in the elutes of both strains when incubated in ΔFHR-1 serum supplemented with FHR-1, compared to incubation in the absence of FHR-1 (**Figure 5**). In summary, these results suggest that FHR-1 attenuates the cofactor activity of FH, leading to an increase in C3 convertase activity and subsequently amplifying the deposition of opsonins C3b and iC3b on *P. aeruginosa.*

**Figure 6:**
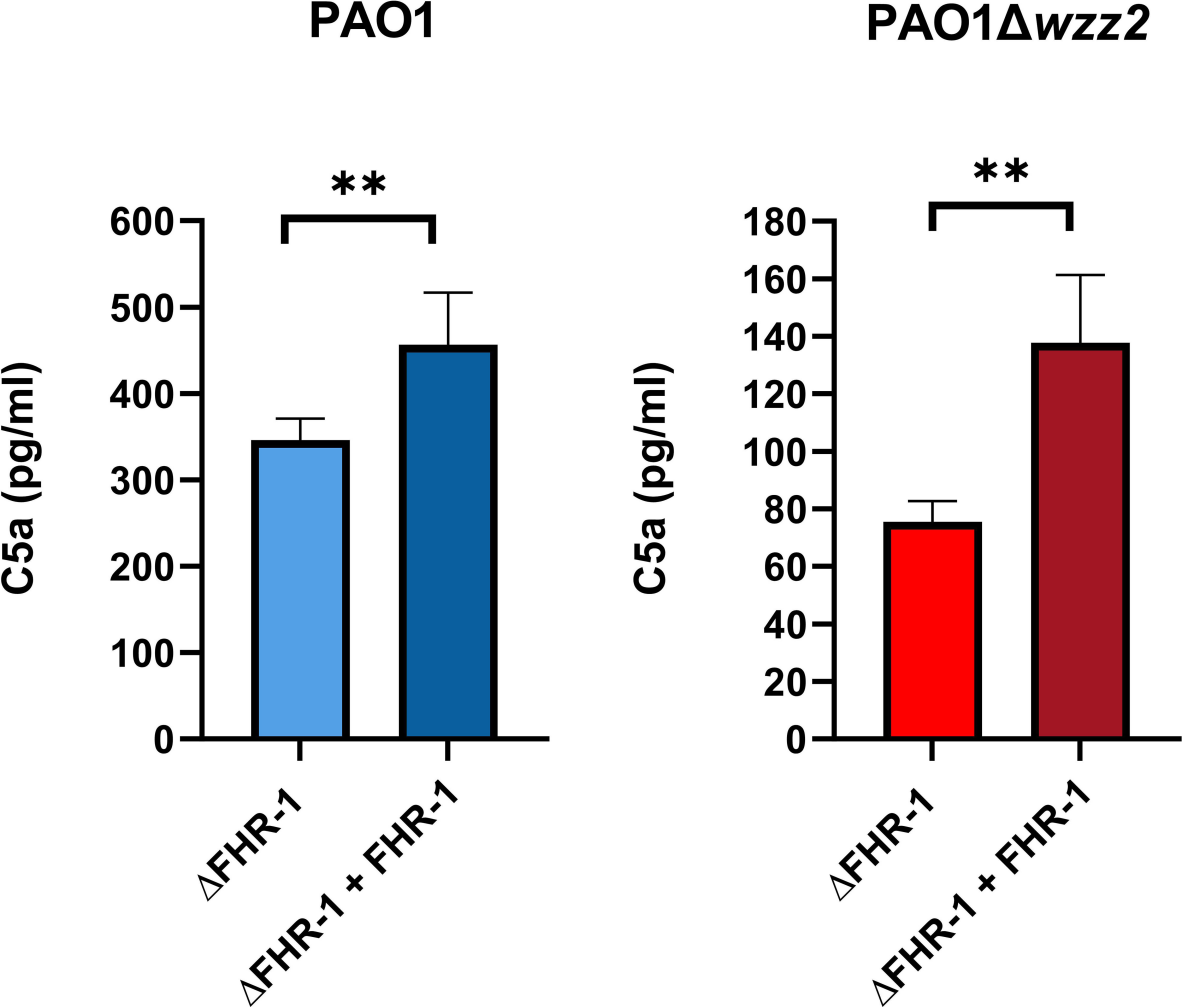
*P. aeruginosa*-induced generation of C5a is enhanced by FHR-1. FHR-1 deficient serum (ΔFHR-1) (25%) supplemented or not with FHR-1 (25 µg/ml) were incubated for 15 min at 37°C with PAO1 or the isogenic LPS deficient mutant PAO1Δ*wzz2*. Levels of C5a were determined by ELISA. Bars represents the mean of at least three independent experiments done in duplicate, and SD is indicated by error bars. Statistical analyses were performed using two-tailed t test; *P* values are indicated on the bars; **P <0.01.

We went one step further and assessed the effect of FHR-1 on the formation of C5a, the most potent chemoattractant molecule involved in the recruitment of neutrophils to the site of infection during the complement activation induced by *P. aeruginosa*. Bacteria cells of both strains, PAO1 and PAO1Δ*wzz2*, incubated in the ΔFHR-1 serum supplemented with FHR-1 induced the formation of higher amounts of C5a compared to those produced in the serum without FHR-1 as demonstrated by ELISA (**Figure 6**). It is noteworthy that PAO1 induced the formation of higher levels of C5a than PAO1Δ*wzz2.* However, the effect of FHR-1 on C5a production was more pronounced with the serum-sensitive strain PAO1Δ*wzz2* where the addition of FHR-1 increased up to 45% the concentration of C5a, while with the serum-resistant strain PAO1 the increment was 24%.

## Discussion

Binding of FHR-1 to *P. aeruginosa* was initially described by Kunert et al. in 2007 (Kunert et al., 2007). They identified EF-Tu as an FHR-1 binding protein of this microorganism. Furthermore, they reported binding of FHR-1 to eight clinical isolates, suggesting that the binding of this host regulator is a common characteristic of *P. aeruginosa* strains. More recently, Hallström et al. demonstrated that LPD is also an FHR-1 binding protein of *P. aeruginosa* (Hallström et al., 2012). Despite both, EF-Tu and LPD being cytoplasmic proteins, they are exposed on the cell surface with “moonlighting” functions such as the binding of the complement regulators FH, FHL-1 and FHR-1 (Kunert et al., 2007; Hallström et al., 2012). In this work, we have identified a novel FHR-1 binding protein, OprG, which is a typical outer membrane protein highly conserved and expressed among *P. aeruginosa* clinical isolates from different origins (Motta et al., 2020; Harmer et al., 2013). Our ligand blot analysis did not detect EF-Tu and LPD as FHR-1 binding proteins, probably because the outer membrane proteins were isolated using a procedure exclusive for typical outer membrane proteins, which would not include EF-Tu or LPD.

The contribution of EF-Tu and LPD to the binding of FHR-1 to *P. aeruginosa* have not been able to be elucidated due to the inability to construct EF-Tu and LPD-deficient mutants due to the essential nature of these proteins. Given that the microbial interactions with host proteins can be mediated by multiple simultaneous mechanisms, we determined the contribution of OprG to FHR-1 binding using a genetic loss-of-function/gain-of-function approach. Our results clearly demonstrate that *P. aeruginosa* binds serum FHR-1 by interacting with the outer membrane protein OprG. However, the OprG-deficient mutant retained 50% of its capacity suggesting that additional FHR-1 binding surface proteins, like perhaps EF-Tu or LPD, may contribute to the interaction of *P. aeruginosa* with FHR-1. Altogether, these findings illustrate that *P. aeruginosa* exploits multiple surface molecules for recruitment of complement regulatory proteins to the bacterial surface.

Our experiments using purified regulatory components FH and FHR-1 demonstrates that FHR-1 competes with FH for the binding to the bacteria. It is likely that this competition arises because FH and FHR-1 share the same bacterial target. Since OprG binds FHR-1, we conducted FH binding experiments on whole cells of the parental strain, the OprG-deficient mutant, and the complemented mutant to investigate whether this outer membrane protein also plays a role in the binding of this regulatory protein. Our results demonstrated that OprG does not significantly contribute to the binding of FH to *P. aeruginosa* (data not shown). Accordingly, the competition between FH and FHR-1 is probably due to other bacterial components. In fact, *P. aeruginosa* displays at least two bacterial proteins, EF-Tu and LPD (Kunert et al., 2007; Hallström et al., 2012), that bind both complement regulatory proteins, FH and FHR-1. However, in experiments using heat-inactivated serum as a source of FH, FHR-1 did not reduce the acquisition of FH by the serum-resistant strain PAO1 or the isogenic serum-sensitive strain PAO1Δwzz2. This observation suggests the existence of other serum factors, which do not require a functional complement system, that interfere in the interaction between FH/FHR-1 with *P. aeruginosa.* Nevertheless, in the competition experiments using a functional human serum and physiological molar ratios of FHR-1/FH, FHR-1 inhibited the binding of FH to *P. aeruginosa*. This result supports the idea that the biological role of FHR-1 in the interaction between the complement system and *P. aeruginosa* is the blockage of the binding of FH to C3b deposited on the bacteria. Therefore, FHR-1 reduces FH-mediated cofactor activity of C3b degradation and C3 convertase decay, as evidenced by the increased deposition of C3 on the microorganism. These results are consistent and supported by previous studies conducted with Group A Streptococcus (Reuter et al., 2010), *Plasmodium falciparum* (Reiss et al., 2018), or DNA and dead cells (Kárpati et al., 2020) showing that FHR-1 is a competitive inhibitor of FH that promotes opsonization.

However, it cannot be ruled out that the increased C3 deposition induced by FHR-1 was due to its direct effect on complement activation function by combining with native C3 (Martin Merinero et al., 2021).

The impact of FHR-1 on complement opsonization was notable on the LPS O antigen-deficient serum-sensitive strain, but negligible on the wild-type serum-resistant strain PAO1, which is among the highest complement-resistant *P. aeruginosa* strains (Pont et al., 2020). It is noteworthy that the addition of FHR-1 to the FHR1-deficient serum had no effect on the killing of the serum-sensitive *P. aeruginosa* strain. Thus, PAO1Δwzz2 was efficiently cleared by the FHR-1 deficient serum, whether complemented or not with FHR-1, probably due to the activation of the classical pathway (data not shown). This observation leads us to hypothesize that recruitment of FHR-1 mainly contributes to increase *P. aeruginosa* opsonization, and this contribution may rely on the specific phenotype of each isolate, consistent with the high variability in survival rates for different *P. aeruginosa* strains in blood (Pont et al., 2020).

Our results also demonstrate that FHR-1 increases C5a production. This observation is contradictory with the inhibitory effect of FHR-1 on the alternative pathway C5 convertase described by Heinen et al. using a FH-depleted serum (Heinen et al., 2009). It is likely that in a serum with FH the regulatory activity of FHR-1 on C5 convertases is insignificant. On the other hand, it has been shown that at physiological concentration, FHR-1 has no intrinsic C5 regulatory activity (Goicoechea de Jorge et al., 2013), suggesting that the enhanced formation of C5a evidenced in our experiments is a logical consequence of the increased C3 convertase activity induced by FHR-1. Altogether, our experimental evidence suggests that FHR-1 enhanced complement activation on the bacterial surface, which results in increased amounts of C3 opsonic fragments and release of C5a that will promote leukocyte recruitment facilitating the clearance of *P. aeruginosa* by opsonophagocytosis.

Given that complement-mediated opsonophagocytosis is the main host defense mechanism to clear *P. aeruginosa* infections, FHR-1 may be considered a host protecting factor. Future studies should focus on the use of *P. aeruginosa Cfhr-1* knock-out mice model of infection to evaluate the role of FHR-1 *in vivo*.

## Data availability statement

The raw data supporting the conclusions of this article will be made available by the authors, without undue reservation.

## Ethics statement

The studies involving humans were approved by the Ethics Review Committee of Human Experimentation of Islas Baleares [Comiteí de eítica de la Investigacioín de las Islas Baleares (CEI-IB)]. The studies were conducted in accordance with the local legislation and institutional requirements. The participants provided their written informed consent to participate in this study.

## Author contributions

AG-A: Investigation, Writing – original draft. HM-M: Investigation, Writing – original draft. MM-B: Investigation, Writing – review & editing. MV: Investigation, Writing – original draft. AD-S: Conceptualization, Investigation, Writing – review & editing. JG: Conceptualization, Writing – review & editing. SC: Conceptualization, Supervision, Validation, Writing – review & editing. SA: Funding acquisition, Investigation, Supervision, Writing – original draft, Writing – review & editing.

## Supplementary material

Supplementary Figure 1 Schematic representation of the human FH, FHL-1 and FHR-1 Functional sites in FH are indicate in brackets on FH. Vertical alignment and colors show Short Consensus Repeats (SCR) homologous to SCRs 6-7 and 18-20 of FH. Sequence homology between FH and FHR-1 is indicated below FHR-1 SCRs. FH-like protein 1 (FHL-1) is a splice variant of FH.

Supplementary Figure 2 SDS-PAGE analysis of the outer membrane proteins of *E. coli* strain S17.1 Λpir and the clone complemented with *oprG.* Outer membrane proteins from *E. coli* strain S17.1 Λpir and the clone complemented with *oprG (*S17.1 Λpir + *oprG)* were isolated, resolved and stained with Coomassie blue.
