# Supplementary figures and images for: Factor H-related protein 1 promotes complement-mediated opsonization of Pseudomonas aeruginosa
Source: Front Cell Infect Microbiol. 2024 Mar 6;14:1328185. doi: 10.3389/fcimb.2024.1328185 (PMC10951071; doi:10.3389/fcimb.2024.1328185)

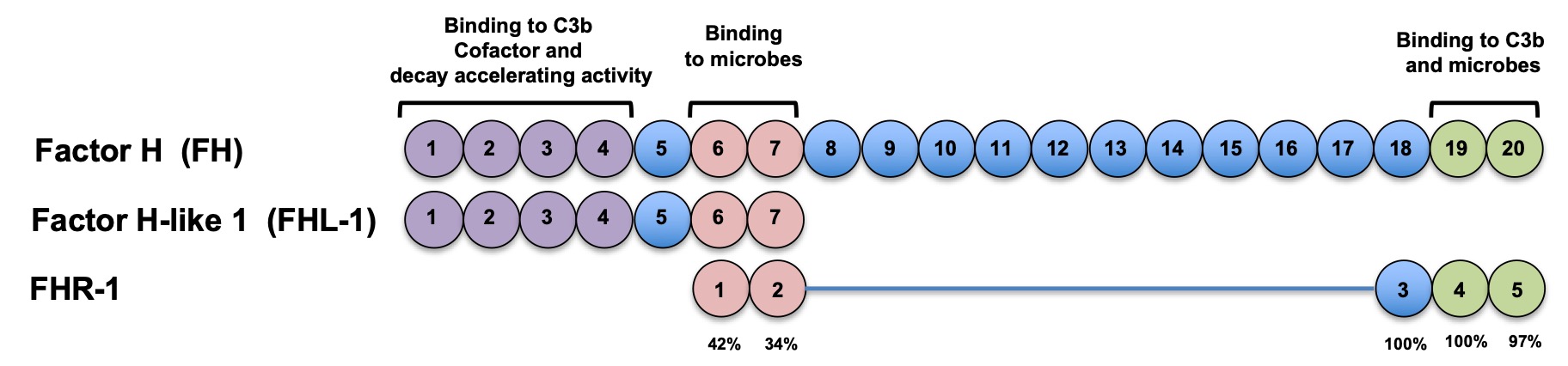

Supplement: Supplementary Figure 1 — Schematic representation of the human FH, FHL-1 and FHR-1 Functional sites in FH are indicate in brackets on FH. Vertical alignment and colors show Short Consensus Repeats (SCR) homologous to SCRs 6-7 and 18-20 of FH. Sequence homology between FH and FHR-1 is indicated below FHR-1 SCRs. FH-like protein 1 (FHL-1) is a splice variant of FH. [file Image_1.jpeg]

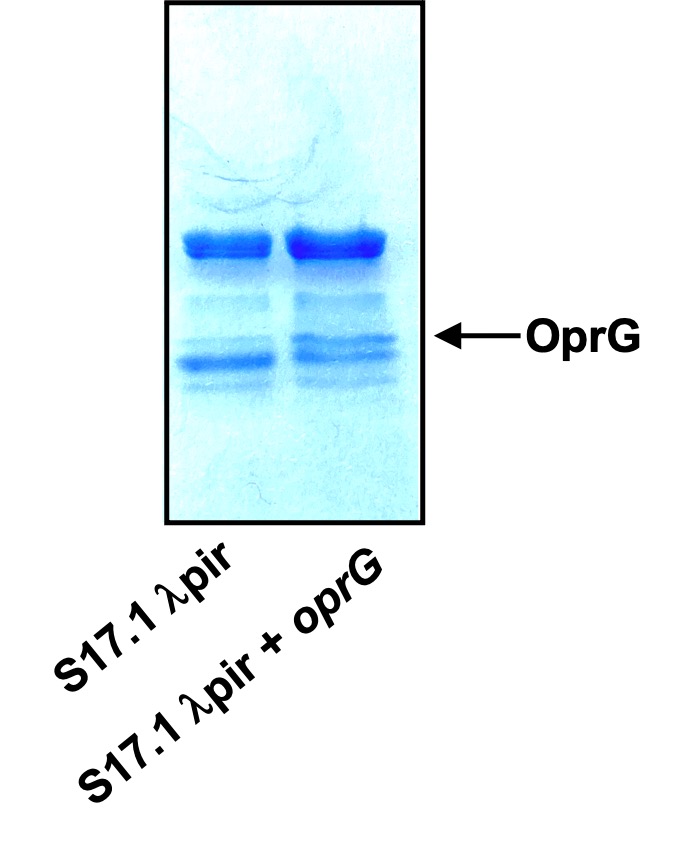

Supplement: Supplementary Figure 2 — SDS-PAGE analysis of the outer membrane proteins of E. coli strain S17.1 Λpir and the clone complemented with oprG. Outer membrane proteins from E. coli strain S17.1 Λpir and the clone complemented with oprG (S17.1 Λpir + oprG) were isolated, resolved and stained with Coomassie blue. [file Image_2.jpeg]
